# Alcoholic Inebriety, from a Medical Standpoint

**Published:** 1883-12

**Authors:** 


*Alcoholic Inebriety, from a Medical Standpoint.* By JOSEPH PARRISH, M.D. Philadelphia: P. Blakiston, Son, & Co. 1883.

It does not appear quite certainly from the Preface whether this book is intended for the Profession or the laity, but apparently it is addressed to both sections of readers; in which case, between the two stools, it goes near to fall to the ground. Its phraseology is in great part too technical to be intelligible to the "general reader" of average education, while its style is too vague to render it of much value in practice to the physician.

The list of contents promised a work of great interest and instruction, and had such chapters as, *e.g.*, "Inebriety and Insanity—How related"; "How to deal with Inebriates"; "Different Alcohols, and their Effects;" and "The Psychology of Inebriety" been adequately and clearly handled, the literature of drunkenness and its cure would have received an addition of serious value. The treatment of these most important subjects, however, is too superficial to leave any distinct impression on one's mind, or to add recognisably to one's information.

The excellent and laudable object of this book is the advocacy of the treatment of Inebriates in Asylums, or Hospitals specially devoted to the purpose, instead of in private houses or Lunatic Asylums. Such institutions have, it is true, met with no very wide-reaching adoption in this country, but that is probably because the entrance into, and residence within them are purely voluntary upon the part of the patient. The case related on pp. 176, 177, enables one to realise how different from those in our own country are the effectual facilities for restraining the Inebriate in the United States; where, therefore, the inhibitory treatment of drunkenness can be more efficiently carried out and attain a higher degree of success.

Like a lady's letter, the pith of this book lies in the postscript, or summary at the end, where, in fifteen propositions, the author gives an abstract of the somewhat nebulous statements contained in the body of the work. Whether after each proposition the reader will feel inclined to write Q.E.D., is a matter of question, though taken collectively they would receive a *general* assent from the medical readers of the work.

As a specimen of our author's style, here is his view as to the origin of the *disease* Inebriety:—

"This disease, however, is not to be regarded as an entity that approaches and invades the human organism from without, but rather as a variation of natural function, having its source in the system itself. It may be implanted somewhere in the complex structure which constitutes the man, by hereditary taint. It may be, by some obscure and undefined qualities coming together, which, by their active and retroactive processes, originate and evolve symptoms that indicate a departure from a healthy standard."

